# A Systematic Review of Cutaneous Hypopigmentation Disorder Associated with Neurologic Involvement

**DOI:** 10.3390/children12121621

**Published:** 2025-11-28

**Authors:** Piero Pavone, Xena Giada Pappalardo, Francesca Scrofani, Raffaele Falsaperla, Francesco Pizzo, Agata Polizzi, Antonio Corsello, Maria Chiara Consentino, Enrico Parano, Martino Ruggieri

**Affiliations:** 1 Department of Clinical and Experimental Medicine, Section of Pediatrics and Child Neuropsychiatry, University Hospital “Policlinico G. Rodolico”, 95100 Catania, Italy; agata.polizzi1@unict.it (A.P.); m.ruggieri@unict.it (M.R.); 2 Institute for Biomedical Research and Innovation (IRIB), Italian National Research Council (CNR), 95123 Catania, Italy; xena.pappalardo@irib.cnr.it (X.G.P.); enrico.parano@cnr.it (E.P.); 3 Department of Biomedical and Biotechnological Sciences (BIOMETEC), University of Catania, 95100 Catania, Italy; francesca.scrofani@studium.unict.it; 4 Unit of Pediatrics and Pediatric Emergency, University Hospital Policlinico “G. Rodolico”, 95123 Catania, Italy; raffaele.falsaperla@unife.it; 5 Postgraduate Training Program in Pediatrics, Department of Clinical and Experimental Medicine, University of Catania, 95100 Catania, Italy; francesco.pizzo@asp.messina.it (F.P.); maria.consentino@asp.sr.it (M.C.C.); 6 Department of Educational Sciences, University of Catania, 95123 Catania, Italy; 7 Pediatric Unit, Department of Clinical, Surgical, Diagnostic and Pediatric Sciences, University of Pavia, 27100 Pavia, Italy; antonio.corsello@unimi.it; 8 Department of Clinical Sciences and Community Health, University of Milan, 20122 Milan, Italy

**Keywords:** clinical sign, cutaneous, hypopigmentation, hypopigmentation disorders, neurological

## Abstract

**Highlights:**

**What are the main findings?**

- Cutaneous hypopigmentation in children spans a wide spectrum of congenital disorders, ranging from localized to diffuse patterns, many of which are associated with neurological involvement.
- This systematic review identifies key clinical features and genetic mechanisms underlying these disorders and provides a practical flowchart to support accurate differential diagnosis.

**What are the implications of the main findings?**

- Early recognition of skin patterns—combined with timely genetic testing and multidisciplinary evaluation—improves diagnostic accuracy and clinical outcomes.
- The structured diagnostic approach helps clinicians distinguish benign findings from conditions requiring urgent neurological assessment and management.

**Abstract:**

**Background/Objectives:** Cutaneous hypopigmentation is a common clinical sign observed in a variety of disorders. It may be congenital or acquired, localized or diffuse, and can range from benign to being associated with systemic conditions, including those affecting the central nervous system. Recognition of the key clinical features associated with each disorder is essential for accurate diagnosis and for differentiating one condition from another. **Methods:** PubMed, Embase, and Scopus databases were used to prepare a systematic review. Search terms were: “congenital”, “cutaneous”, “localized”, “diffuse”, “hypopigmentation” and “neurological disorders/signs”. The focus was on congenital cutaneous hypopigmentation, distinguishing between localized and diffuse presentations, with emphasis on neurological involvement. Peer-reviewed articles, case series, and original studies with neurological manifestations of the disease were reviewed. Cutaneous hypopigmentation disorders associated with diffuse cerebral involvement include syndromes such as Chediak–Higashi, Griscelli, Elejalde, Cross, Tietz albinism-deafness, Prader–Willi, Angelman, and several congenital metabolic disorders. In contrast, the ‘localized’ group comprises syndromes such as Waardenburg, Incontinentia pigmenti, and the phacomatoses, including hypomelanosis of Ito and tuberous sclerosis complex. **Results:** Overlapping phenotypes and genetic heterogeneity lead to diagnostic challenges and potential errors suggesting multiple specialists. The main clinical features of each disorder are discussed, with particular attention paid to neurological signs. A practical flowchart is provided to help distinguish between ‘diffuse’ and ‘localized’ forms with neurological involvement. **Conclusions:** Early identification of cutaneous features, followed by comprehensive clinical and instrumental evaluation, enables timely and accurate diagnosis, ultimately improving the clinical outcomes for affected children.

## 1. Introduction

Cutaneous hypopigmentation is a clinical sign that may be congenital or acquired and involve either unilateral or bilateral localization. It can present as a common, isolated, and benign finding, but it may also indicate a wide spectrum of disorders affecting the brain and other organ systems. Melanocytes, which produce skin pigment, originate from neural crest cells and reside in the epidermis [1]. They create melanin within melanosomes, which are then transferred to keratinocytes [2]. Pigmentation relies on enzymes, structural proteins, and transport proteins [3]. Hypopigmentation can arise from issues with melanocyte number/function, melanosome defects, or transfer problems [1]. In children, hypopigmented skin disorders can also be categorized based on the age of onset, early or later in childhood, the extent of skin involvement, diffuse (generalized) or localized, and the underlying etiological factors, including genetic contributions that influence these cutaneous events [4,5]. Genetic cutaneous hypopigmentation can result from mutations that disrupt the complex pathways involved in melanocyte development and function. These mutations may affect various stages of melanocyte biology, including melanoblast development and migration, as seen in disorders such as piebaldism, Waardenburg syndrome (WS), and Tietz albinism-deafness syndrome (TADS); melanin synthesis, as observed in oculocutaneous albinism (OCA); and melanosome formation and transfer to keratinocytes, as occurs in Hermansky–Pudlak syndrome (HPS), Chediak–Higashi syndrome (CHS), and Griscelli syndrome (GS) [6]. The pattern of skin is variable and may appear as single, patchy, or scattered lesions. Shapes can include linear, triangular, circular, segmental, or leaf-like forms.

In some cases, the distribution may follow specific patterns, such as along the lines of Blaschko [7]. While cutaneous hypopigmentation is often a common and benign finding in childhood, it can sometimes indicate underlying neurological disorders, prompting referral to pediatric neurologists. Dermatologists, neurologists, neuropediatricians, ophthalmologists, neuropsychiatrists, psychologists, and geneticists play a critical role not only in establishing the diagnosis but also in managing the long-term clinical course of these disorders. Early genetic diagnosis is essential, particularly in cases of mosaicism in pigmentary mosaic disorders, and should be accompanied by appropriate genetic counseling for affected families.

This study provides an updated overview of childhood disorders characterized by congenital cutaneous hypopigmentation, with emphasis on those that include neurological involvement. Its aim is to offer practical clinical guidance for evaluating patients whose hypopigmented skin findings are associated with neurologic features. Key clinical signs are summarized, highlighting the role of genetic testing and multidisciplinary approaches in achieving early and accurate diagnosis, as well as guiding effective management. 

## 2. Materials and Methods

**Figure 1:**
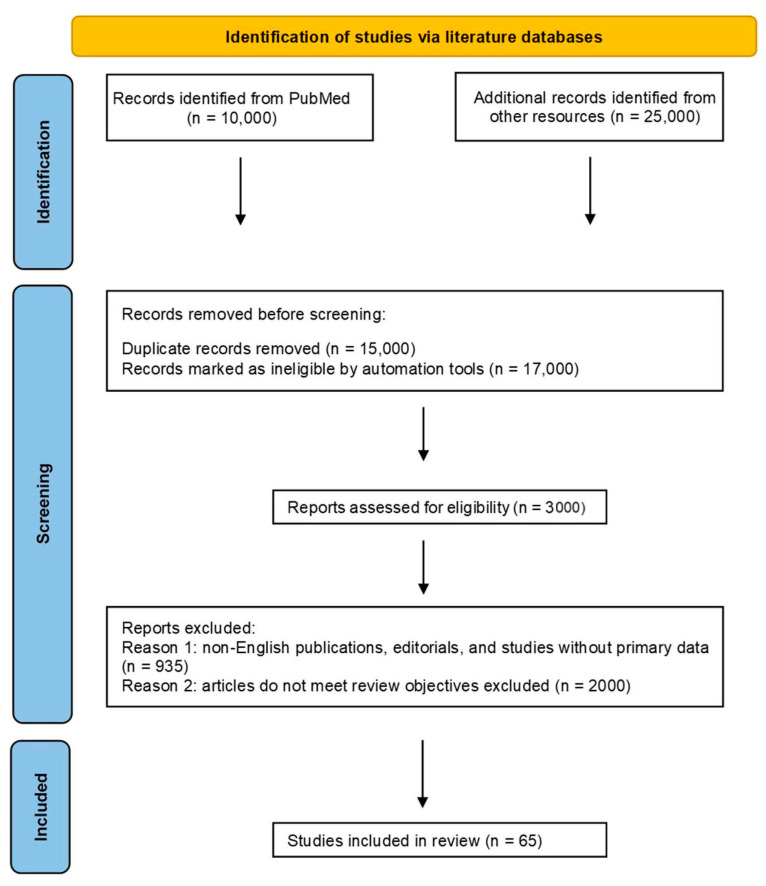
Schematic illustration of the PRISMA flowchart used in the present review.

The present work was prepared in accordance with the Preferred Reporting Items for Systematic Reviews and Meta-Analyses (PRISMA) statement (Figure 1), see File S1 [8]. It examined research from 2000–2023 using databases like MEDLINE, Embase, PubMed, Cochrane Central and Scopus. Search terms were focused on genetic disorders, hypopigmentation, and related conditions. Relevant studies were retrieved from PubMed (n = 10,000) and additional databases and sources (n = 25,000). Inclusion criteria referred to subjects < 18 years, congenital vs. acquired, requirement for neurologic outcomes. After removal of duplicate records (n = 15,000) and exclusion of articles that did not meet the inclusion criteria or were outside the scope of the review (n = 17,000), 3000 studies remained for full-text assessment. Of these, 65 studies met the eligibility criteria and were included in the final qualitative synthesis, focusing specifically on the neurological features associated with cutaneous hypopigmentation disorders. The present review protocol was not registered in any database.

## 3. Results

A list of the most common acquired hypopigmentation disorders [4] is reported in Table 1.

Congenital cutaneous hypopigmentation disorders with known neurological involvement and systemic manifestations are categorized into two groups: diffuse and localized. The diffuse groups include Angelman syndrome (AS), Cross syndrome (CS), CHS, Elejalde syndrome (ES), GS, and several inborn errors of metabolism such as phenylketonuria, homocystinuria, histidinemia, and Menkes syndrome. The localized group includes Incontinentia pigmenti (IP) (stage IV), Hypomelanosis of Ito (HI), and Tuberous sclerosis complex (TSC), WS. Detailed classifications are shown in Table 2a,b.

## 4. Discussion

Diagnosing skin hypopigmentation in children involves distinguishing between congenital (diffuse or localized) and acquired forms, considering family history, and performing systemic and genetic testing. Collaboration among specialists is crucial for accurate diagnosis and treatment, especially when neurological or other systemic issues are present.

### 4.1. Congenital Diffuse Cutaneous Hypopigmentation Disorders and Neurological Impairment

CHS (MIM:214500) is a rare autosomal recessive disorder caused by mutations in the *CHS1*/*LYST* gene, located on chromosome 1q43. The syndrome is characterized by silvery hair, oculocutaneous albinism, easy bruising, impaired function of natural killer cells, and recurrent pyogenic infections. Affected children may also develop features of severe hemophagocytic syndrome (HPS), resulting from uncontrolled activation of T-cells and macrophages [4,9]. In CHS, massive cytoplasmic inclusions, both lysosomal and non-lysosomal, in granule-containing cells likely contribute to impaired cellular function. Abnormally large intracytoplasmic granules in white blood cells and bone marrow serve as key diagnostic markers. Alongside skin findings, affected children often present with recurrent severe infections, bleeding tendencies, anemia, lymphadenopathy, and hepatosplenomegaly [10,11]. Morbidity and neurological complications in CHS stem from recurrent infections or an accelerated lymphoproliferative phase impacting vital organs, with some patients dying within the first decade. Survivors may develop neurodegenerative symptoms in early adulthood, such as spinocerebellar ataxia, sensory neuropathies, and cognitive decline [10,11,12]. Hematopoietic stem cell transplantation (HSCT) is particularly effective before the accelerated phase of the disorder. Genetic analysis of LYST/CHS1 genes is diagnostic for CHS. The treatment for hematological and immune deficiencies involves allogeneic hematopoietic stem cell transplantation, which should be performed as soon as the diagnosis is confirmed [10].

GS is an autosomal recessive disorder characterized by light-colored skin, silvery-gray hair, and large pigment clumps within the hair shafts. Three distinct types of GS have been identified. GS type 1 (GS1; MIM:214450) is associated with mutations in the *MYO5A* gene on chromosome 15q21, which encodes the organelle motor protein myosin-VA [13,14]. The protein functions to regulate organelle transport in both melanocytes and neuronal cells. Patients with GS type 1 typically present with partial albinism, immunodeficiency, recurrent febrile episodes, hepatosplenomegaly, severe cytopenia, and primary neurological impairments such as developmental delay (DD) and ID. The clinical features closely resemble those seen in Elejalde syndrome, with which GS type 1 has been associated. *MYO5A* results are positive and support a diagnosis of GS1. GS type 2 (GS2; MIM:607624) is caused by mutations in the *RAB27A* gene, located on chromosome 15q21. This gene encodes Rab27a, a small GTPase protein that plays a critical role in the regulated secretory pathway within cells. Affected children exhibit albinism alongside severe clinical manifestations resulting from hemophagocytic syndrome (HPS), which affects multiple organs and can lead to serious complications including neurological involvement [15,16]. Minimal first line diagnostic test includes results of complete blood cell (CBC) count, smear, platelet function, and immune globulin. Genetic analysis of the *RAB27A* gene is diagnostic per GS2. Bone marrow transplantation is the recommended treatment. GS type 3 (MIM:609227) is the mildest form of the syndrome, characterized by light skin and silvery hair without neurological symptoms. GS type 3 results from mutations in *MLPH*, the gene encoding melanophilin. Melanophilin forms a protein complex with Rab27a and myosin-Va, facilitating melanosome transport within melanocytes [14]. The genetic analysis of the *MLPH* gene is diagnostic for GS3.

ES (MIM:256710) also known as neuroectodermal melanolysosomal disease, is an autosomal recessive disorder characterized by silvery hair and severe central nervous system dysfunction. Originally described by Elejalde et al. [17] in three siblings with a shared ancestry, the syndrome manifests with severe dysfunction of the central nervous system impairment and silver-leaden hair coloration. Affected children exhibit abnormal melanosomes and melanocytes. Additionally, abnormal lysosomal inclusion bodies have been identified in fibroblasts, bone marrow histiocytes, and lymphocytes [17]. Clinically, patients present with bronze skin after sun exposure, profound cognitive impairment, seizures, hypotonia, and reduced or absent voluntary movements. Although ES shares clinical features with GS type 1, immunological issue are less common in ES [17,18,19,20,21,22]. Treatment remains symptomatic and supportive. 

CS (MIM:257800) also known as oculocerebral syndrome with hypopigmentation or Kramer syndrome, is a rare genetic disorder inherited in an autosomal recessive pattern. It is characterized by a combination of ocular and cutaneous hypopigmentation along with neurological abnormalities. The condition was first described by Cross et al. [23]. This syndrome was identified in two males and two females from an inbred Amish family, all showing cutaneous hypopigmentation, severe eye anomalies, growth delay, and early-onset neurological issues such as profound intellectual disability, spastic tetraplegia, and involuntary movements. Affected infants often present with silvery-gray hair, very light skin, and marked photosensitivity. The clinical spectrum has since broadened to include multiple congenital anomalies across various organ systems. Notably, developmental defects of the central nervous system have been reported, including cystic malformations of the posterior fossa consistent with Dandy-Walker malformation, ref. [24] as well as vertebral fusion at the L4–L5 level [25]. Additional manifestations include dolichocephaly, an underdeveloped diaphragm, and a range of ocular anomalies such as cloudy corneas, optic atrophy, cataracts, microphthalmia, and nystagmus [26]. Urinary tract abnormalities, bilateral hernias, and cardiac anomalies, such as focal interventricular septal hypertrophy, have also been reported [26]. Affected children may develop abnormally enlarged gums and gingival fibromatosis, characterized by a pink, leathery appearance with small, pebble-like bumps on the surface [27]. Chabchoub et al. [25] identified a de novo interstitial deletion at 3q27.1–q29 on the paternal chromosome. Management of affected individuals includes symptom-specific and supportive therapies, along with specialized remedial education.

TADS (MIM:103500) is a genetic condition characterized by bilateral congenital sensorineural hearing loss and generalized hypopigmentation of the eyes, iris, and hair. It follows an autosomal dominant inheritance pattern and is caused by mutations in the melanocyte-inducing transcription factor (*MITF*) gene, located on chromosome 3p12. This gene plays a critical role in regulating melanin biosynthesis in human melanocytes and contributes to the pigmentation of hair, eyes, and skin [28,29]. Melanocytes have been identified in the inner ear, where they are believed to play a crucial role in hearing. Mutations in the *MITF* gene are thought to contribute to bilateral sensorineural hearing loss. Affected individuals typically present from infancy with very pale skin, but without heterochromia of the irides. Hair color often changes over time, becoming blond or red [30].

Prader–Willi syndrome (PWS; MIM:176270) is a complex genetic disorder caused by the loss of expression of paternally inherited genes within the PWS critical region on chromosome 15q11–q13 [31]. PDS presents with hypotonia and feeding difficulties in infancy, followed by obesity and delayed puberty. Common features include vision problems, a distinctive face, shortened limbs, skeletal anomalies, scoliosis, and reduced pain sensitivity [32]. Neurological involvement includes DD and cognitive impairment, often managed with anticonvulsant therapy [33,34]. Cutaneous, hair, and eye hypopigmentation observed in patients with PWS has been associated with a deletion of oculocutaneous albinism type 2 (*OCA2*) [4] gene on chromosome 15. In PWS, skin and mucosal picking, often affecting the nose, rectum, and genitals, is a common dermatologic feature. The clinical course progresses through defined phases, starting prenatally with reduced fetal movements and growth restriction, followed by neonatal hypotonia, feeding difficulties, and later, unexpected growth with hyperphagia and weight gain. Management requires a multidisciplinary approach, including early nutritional support, endocrine treatment, developmental and educational interventions, and ongoing behavioral therapy to improve long-term outcomes.

AS (MIM:105830) is a neurodevelopmental disorder primarily affecting the nervous system, most often caused by a de novo mutation in the maternal *UBE3A* gene on chromosome 15q11–q13. About 70–75% of cases result from a maternal deletion in this region. Less common causes include paternal uniparental disomy, imprinting defects, or point mutations in the maternal *UBE3A* allele [35]. AS is characterized by craniofacial differences, developmental delays, speech impairment, intellectual disability, microcephaly, and drug-resistant epilepsy. Patients often display a happy demeanor, motor issues, and sleep disturbances. Additional features include eating problems, obesity risk, and hypopigmentation [2,35,36]. Some cases combine with *OCA2*, leading to white skin, golden hair, and pale irises [37,38]. Treatment typically includes occupational, physical, and speech-language therapy. For seizure management, clobazam or levetiracetam is recommended as first-line therapy. The use of valproate should be avoided, as it may worsen tremors and lead to regression of motor skills [39,40]. Clinical trials are currently underway for the transplantation of autologous hematopoietic stem cells genetically modified with a lentiviral vector to express *UBE3A* [41].

Certain inborn errors of metabolism, such as phenylketonuria, histidinemia, homocystinuria, and Menkes disease, can cause cutaneous discoloration, especially when left undiagnosed or untreated.

Phenylketonuria (PKU; MIM:261600) is primarily caused by missense mutations in the gene encoding phenylalanine hydroxylase (PAH), the enzyme responsible for the hydroxylation of phenylalanine to produce tyrosine. The widespread implementation of neonatal screening programs for PKU and other metabolic conditions has enabled early diagnosis and timely dietary treatment, allowing many patients to remain asymptomatic. PKU is primarily caused by missense mutations in the PAH gene, impairing the conversion of phenylalanine to tyrosine. Newborn screening enables early diagnosis and dietary management, often preventing symptoms. Classic PKU may present with a musty odor, developmental delay, intellectual disability, seizures, microcephaly, skin rash, and hypopigmentation due to reduced melanin synthesis [42]. Positive neonatal heel prick screening test plasma with diagnosis confirmed by amino acids analysis and PAH gene mutations for PKU.

Histidinemia is caused by a deficiency of histidase, the enzyme responsible for the irreversible, non-oxidative deamination of histidine to urocanic acid, primarily in the liver and skin. Individuals with this metabolic disorder exhibit elevated blood levels of histidine and increased excretion of histidine, imidazole pyruvic acid, and other imidazole derivatives in the urine. Although histidinemia is generally considered a benign condition, neurological impairments have been reported, likely due to additional pathological factors [43]. This disorder may also contribute to cutaneous discoloration by interfering with melanin synthesis [4].

Homocystinuria is caused by a deficiency of cystathionine β-synthase, an enzyme involved in the metabolism of homocysteine. The clinical manifestations are diverse and may affect multiple organ systems. Ocular abnormalities include ectopia lentis and/or severe myopia. Skeletal features often resemble those of Marfan syndrome and include excessive height, elongated limbs, scoliosis, and pectus excavatum. Vascular involvement, particularly thromboembolic events, represents a major clinical concern. Central nervous system manifestations may include DD and ID. Cutaneous findings can include malar flushing, extremely fine, fragile, and thin skin, as well as hypopigmentation [44].

Menkes Disease (MIM:309400) is an X-linked recessive disorder that primarily affects males. It is caused by mutations in the ATPase copper transporting alpha (*ATP7A*) gene, most commonly intragenic mutations or partial gene deletions. The *ATP7A* gene encodes an energy-dependent transmembrane protein responsible for delivering copper to copper-dependent enzymes and exporting excess copper from cells [45,46,47]. Use of subcutaneous or intravenous administering of copper histidine component is the goal of treatment. Menkes disease in infancy may present with jaundice, hypothermia, and hypoglycemia. Hallmark signs include sparse, brittle, kinky hair and sagging facial features. Developmental regression, hypotonia, and epileptic seizures typically begin around 5–6 months, along with skeletal abnormalities and osteoporosis [45,46,47]. First line diagnostic exam includes copper value for Menkes syndrome. A favorable response to copper-histidine treatment is associated with early, ideally prenatal, diagnosis. Vairo et al. [48] have recommended prenatal screening in families with a known history of Menkes disease by analyzing fetal plasma catecholamine levels. *ATP7A* testing is positive, consistent with the Menkes diagnosis. If results are indicative of the disorder, initiating copper-histidine therapy in the neonatal period may improve survival and help prevent severe neurological complications. 

### 4.2. Congenital Localized Cutaneous Hypopigmentation Disorders and Neurological Impairment

WS (MIM:193500) refers to a group of genetic disorders inherited in an autosomal dominant manner. The condition is caused by the loss or dysfunction of pigment-producing cells in the eyes, skin, hair, and the stria vascularis of the cochlea. Clinical manifestations vary among individuals and may include craniofacial dysmorphisms, varying degrees of congenital hearing loss, and pigmentation abnormalities [49]. Major and minor diagnostic criteria have been established for the clinical identification of WS. Major criteria include heterochromia of the irises, sensorineural hearing loss, a white forelock, lateral displacement of the inner canthi (telecanthus), and having a first-degree relative with WS. Minor criteria include a broad nasal root, white skin macules or patches, synophrys (medial eyebrow fusion), premature graying of scalp hair, and hypoplasia of the nasal alae. A clinical diagnosis of WS typically requires the presence of either two major criteria or one major criterion in combination with at least two minor criteria [49,50]. WS is classified into four recognized types, distinguished by their clinical features and underlying genetic causes. It is worth noting that the inclusion of WS among syndromes involving both cutaneous and neurological manifestations remains debated, as such associations have been inconsistently and infrequently reported [7]. The treatment of WS focuses primarily on early diagnosis and timely management of hearing impairment to prevent cognitive and behavioral complications. When appropriate, surgical intervention, such as cochlear implantation, may be considered to restore hearing function. Sun protection is also recommended, particularly for individuals with hypopigmented skin. The key clinical features of the four recognized subtypes of WS are summarized below. WS type 1 (WS1; MIM:193510) may present with craniofacial anomalies, including dystopia canthorum, defined as lateral displacement of the inner canthi with a normal interpupillary distance, along with a prominent nasal root, short philtrum, retrognathia, and medial eyebrow fusion (synophrys). A white forelock and premature graying of the hair are commonly observed. Cutaneous depigmented patches and pigmentary abnormalities of the iris, such as partial or complete heterochromia, may also be present [1,4,6,49,50]. The diagnosis of GS1 has been confirmed by mutations in the paired box 3 (*PAX3)* gene, located on chromosome 2q35–q37.3. WS type 2 (WS2; MIM:193510) present with normally positioned canthi, premature graying of the hair, depigmented white skin patches, heterochromia iridis, and congenital sensorineural hearing loss [49]. The syndrome has been associated with mutations in the melanocyte inducing transcription factor (*MITF)* gene, located on chromosome 3p12, confirming the diagnosis of WS2. Additionally, mutations in the snail family transcriptional repressor 2 (*SNAI2)* gene (also known as the *SLUG* gene), located on chromosome 8q, have also been reported [49]. WS type 3 (WS3; MIM:148820) exhibits features similar to those seen in WS1, along with additional musculoskeletal congenital anomalies. These include hypoplasia of the upper limb muscles, flexion contractures, fusion of the carpal bones, and syndactyly. Some individuals may also present with microcephaly and intellectual disability. WS3 is caused by mutations in the *PAX3* gene. WS type 4 (WS4; MIM:277580) shows variable clinical manifestations of classical WS have been reported in association with congenital megacolon (Hirschsprung disease). Affected individuals may carry a heterozygous mutation in the SRY-related HMG-box (*SOX*) gene family, or a homozygous mutation in either the endothelin 3 (*EDN3*) gene, which encodes a peptide ligand, or its receptor gene *EDNRB* [6,51,52,53]. Therefore, *SOX*, *EDNRB* and *EDNR* are indicative for the WS4 diagnosis.

IP (MIM:308300). This condition is an X-linked dominant genodermatosis characterized by a complex and variable range of clinical manifestations affecting multiple organ systems, including the skin, hair, teeth, nails, eyes, and central nervous system. It occurs almost exclusively in females. In most cases, the disorder results from a de novo mutation in the inhibitor of kappa B kinase gamma (*IKBKG)* gene, located on chromosome Xq28. *IKBKG* plays a key role in activating nuclear factor kappa-light-chain-enhancer of activated B cells, a transcription factor involved in numerous cellular pathways, including apoptosis, cell cycle regulation, inflammation, and immune responses [54]. Skin manifestations evolve through four stages: Stage I (vesicular), characterized by blistering that appears at birth or within the first four months of life; Stage II (verrucous), a wart-like rash that develops and may persist for several months; Stage III (hyperpigmented), swirling macular hyperpigmentation that emerges around six months of age and can persist into adulthood; and Stage IV (hypopigmented/atrophic), linear hypopigmentation with atrophic changes, associated with a reduction in melanocytes in the basal layer [55]. Alopecia, hypodontia, dystrophic nails, and ocular retinal complications are common features. Neurological manifestations occur in approximately 30% of patients with IP, with most neurological symptoms presenting during the neonatal or early infantile period. Seizures and infantile spasms are reported in 20–30% of cases and appear to correlate with the extent of cerebrovascular damage. Brain MRI findings may include periventricular and subcortical white matter involvement, hemorrhagic changes, hypoplasia of the corpus callosum, as well as cerebral and cerebellar atrophy [56]. Treatment involves standard care for skin blistering and infections, prevention of retinal detachment, and neurological management of seizures, spasticity, and focal brain deficits [54,55].

**Figure 2:**
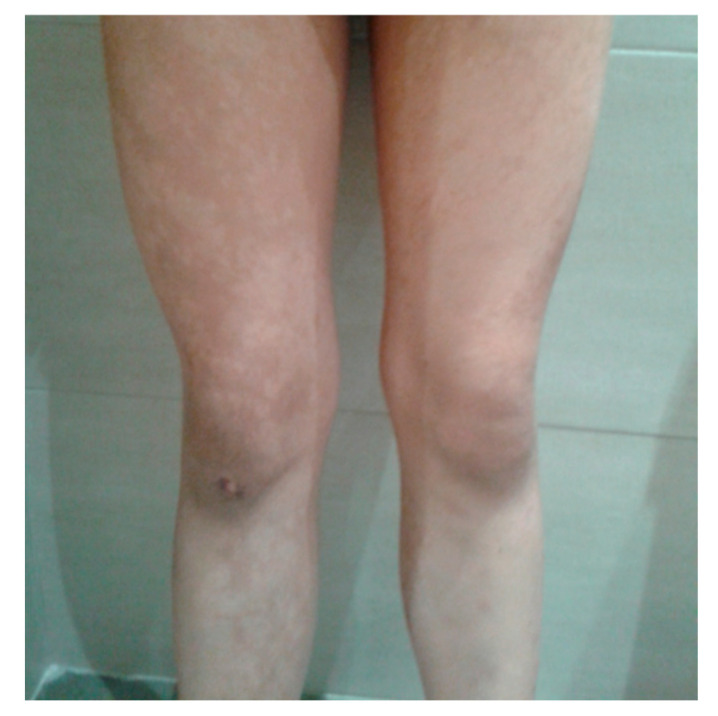
Legs with typical nevoid hypopigmentation in a 14-year-old girl affected by HI.

HI (MIM:00337) is the third most common neurocutaneous disorder, following neurofibromatosis type 1 and tuberous sclerosis complex. The terms neurocutaneous disorders and phacomatoses are often used interchangeably to describe a heterogeneous group of conditions that involve congenital pigmentary or vascular abnormalities of the skin, and are commonly associated with congenital anomalies of the central and/or peripheral nervous system, tumors, and variable ocular abnormalities [57]. Clinical features may also affect multiple other organs and systems, including the heart, vessels, lungs, kidneys and bones [57]. The broad range of clinical manifestations in HI is attributed to complex interactions between intra- and extra-neuronal signaling pathways, including receptor-to-protein and protein-to-protein cascades involving multiple genes [58]. HI typically presents with linear nevoid hypopigmentation following the lines of Blaschko, primarily affecting the limbs and trunk. These distinct areas of pigmentation reflect the distribution of two different cell lines within the individual, each with varying pigment-producing potential [57]. The condition is caused by a clone of skin cells with reduced melanin-producing capacity. Chromosomal aberrations may disrupt the loci of genes involved in pigmentation, contributing to the observed hypopigmented patterns [57]. In this disorder, cutaneous anomalies are typically present at birth or during infancy. DD and epilepsy are among the most common and earliest reported symptoms [58]. Pavone et al. [59] reported five patients with HI who exhibited characteristic skin anomalies (Figure 2) and early-onset epileptic seizures, despite having normal brain MRI findings. Treatment of HI is directed toward the clinical manifestations. Skin lesions generally do not require specific therapy. Peripheral avascular retina lesions are treated with laser photocoagulation. The goal of the ophthalmologic symptoms is to prevent retinal detachment or vitreous hemorrhage secondary to retinal neovascularization. Special education support and anticonvulsant medication may be beneficial when indicated.

**Figure 3:**
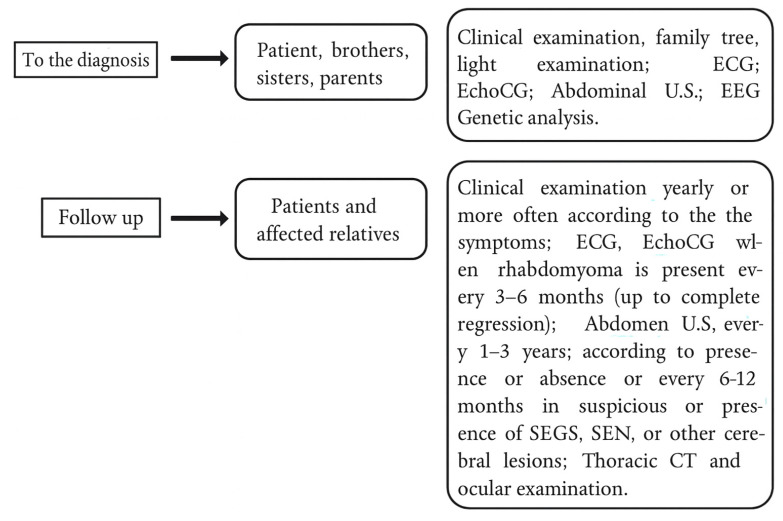
Diagnostic evaluation and follow-up protocol for children with Tuberous Sclerosis Complex (TSC) at the Pediatric Department, University of Catania, Italy. Abbreviations: ECG (electrocardiogram); EchoCG (echocardiography); U.S. (ultrasound); EEG (electroencephalogram); MRI (magnetic resonance imaging); CT (computed tomography); SEGS (subependymal giant-cell astrocytomas); SEN (subependymal nodules).

TSC is an autosomal dominant, multisystem genetic disorder characterized by a wide range of clinical manifestations affecting various organs, including the skin, brain, kidneys, and lungs. Central nervous system tumors are the leading cause of morbidity and mortality in affected individuals. TSC is caused by mutations in either the *TSC1* (MIM:605284) or *TSC2* gene (MIM:191092) [60,61]. The *TSC1* gene codes for hamartin, and *TSC2* codes for tuberin. Mutations in these genes disrupt their function as tumor suppressors, leading to abnormal cell growth and development via dysregulation of the mTOR pathway [60,61,62,63]. Genetic analysis may fail to detect mutations in some patients who meet the criteria for TSC [62,63]. Positive *TSC1* and *TSC2* results are diagnostic for TSC types 1 and 2, respectively. Early signs of TSC on the skin include hypopigmentation (“ash leaf spots,” “confetti-like” lesions, or white epidermal nevi) [1,4,60]. Self-adjusting file (SAF) and dermatoscopy are useful to better define color and limits of the cutaneous lesions to TSC. Individuals with more than three of these spots are at high risk for TSC [57,61,64]. Other skin features include facial angiofibromas, shagreen patches, fibrous cephalic plaques, and ungual fibromas (Koenen tumors) [64]. Brain tumors, such as cortical tubers, subependymal nodules (SEN), and subependymal giant cell astrocytomas (SEGA), are common and require early diagnosis [61,62,64,65]. TSC brain lesions can cause serious problems, especially neurological ones. Early symptoms include infantile spasms, often linked to cognitive regression and DD [66]. Epileptic seizures, often focal or bilateral tonic–clonic, are common in TSC. Early recognition and management often lead to good seizure control. Other neurological issues may include behavioral problems, emotional disturbances, learning difficulties, cognitive impairment, and autism spectrum disorder [64]. Kidney involvement is common in TSC, with angiomyolipomas being the most frequent lesions. Other possible findings include renal cysts, oncocytomas, and, rarely, malignant angiomyolipomas [64,65]. The most common cardiovascular manifestation of the TSC is the cardiac rhabdomyoma [66,67]. Cardiac rhabdomyomas are usually harmless and disappear within three years [67]. Pulmonary complications include lymphangioleiomyomatosis (primarily in women) and multifocal micronodular pneumocyte hyperplasia (MMPH) [68]. Eye problems often involve benign retinal hamartomas, but complications like retinal detachment can occur [69]. Other features include hypopigmented skin lesions (reported in ~39% of cases [64]), bone changes, liver tumors, and dental anomalies [61]. The diagnostic criteria for TSC were recently revised by Northrup et al. [64] distinguishing major and minor clinical features. Major criteria include: ≥3 hypomelanotic macules (≥5 mm), ≥3 facial angiofibromas or a fibrous cephalic plaque, ≥2 ungual fibromas, a shagreen patch, multiple retinal hamartomas, cortical tubers and/or radial migration lines, ≥2 subependymal nodules, subependymal giant cell astrocytoma, cardiac rhabdomyoma, lymphangioleiomyomatosis, and ≥2 renal angiomyolipomas. Minor criteria include: “confetti” skin lesions, ≥3 dental enamel pits, ≥2 intraoral fibromas, a retinal achromic patch, multiple renal cysts, nonrenal hamartomas, and sclerotic bone lesions. Regarding the treatment, pre-symptomatic anticonvulsant therapy with vigabatrin is recommended as soon as epileptiform activity is detected on EEG, even before clinical seizures emerge [70,71]. Vigabatrin is particularly effective in managing infantile spasms [72,73]. mTOR inhibitors, such as sirolimus and everolimus, have demonstrated efficacy in controlling various seizure types and improving other TSC-related manifestations [74,75,76,77,78,79]. Cannabidiol is a potential adjunct therapy, and vagus nerve stimulation may be considered in selected cases with TSC-associated epilepsy [80,81]. For patients with drug-resistant, focal epilepsy, early surgical intervention remains a key therapeutic option [82]. Diagnostic and follow-up algorithms in TSC patients, from childhood to young adulthood, have been developed and implemented by the Pediatric Department of Catania University are reported [83] in Figure 3.

## 5. Limitations

This review examined disorders characterized by congenital cutaneous hypopigmentation accompanied by neurological features, with the aim of outlining key clinical characteristics to aid diagnostic evaluation. However, substantial phenotypic overlap among these conditions can complicate the establishment of a definitive and specific diagnosis. Advances in genetic diagnostic technologies, both current and forthcoming, are expected to enhance the classification of these syndromes and facilitate earlier and more accurate clinical intervention. Furthermore, publication bias is also related to heterogeneous clinical expression of the disorder and with some overlapping of clinical features presented by each of the affected individuals.

## 6. Future Directions

As clinical medicine increasingly integrates artificial intelligence (AI), future efforts in the evaluation of cutaneous hypopigmentation disorders with neurologic involvement should explore the potential of AI-driven diagnostic support. Recent studies have demonstrated the effectiveness of AI models in dermatology for identifying and classifying skin conditions [84,85,86] highlighting an opportunity to extend these tools to disorders of pigmentation. A practical future direction would involve linking clinical workflows with an AI system capable of analyzing standardized skin photographs, automatically identifying and counting hypopigmented lesions, and highlighting them for clinician review. Such a tool could also generate preliminary risk tiers (e.g., urgent vs. routine evaluation) and translate visual findings into structured terminology that interfaces directly with the electronic health record. In turn, this could prompt context-appropriate next steps—such as EEG, genetic testing, or metabolic screening—thereby supporting earlier recognition, streamlined triage, and more precise diagnostic pathways.

## 7. Conclusions

Cutaneous hypopigmentation can indicate underlying conditions, potentially signaling serious systemic disorders, especially affecting the brain. Neurologic assessment must be ratified through clinical history and physical examination, laboratory and neuroradiological exams including brain MRI and CT scan, electroencephalogram carried out when awake and during sleep, and developmental tests and genetic analysis are necessary to make a rapid and correct diagnosis. Diagnosis can be challenging, but genetic testing is improving accuracy. Early recognition and interventions can enhance outcomes and quality of life for affected children.

## Figures and Tables

**Table 1:** Common acquired cutaneous hypopigmentation in children.

Nutritional deficiency (insufficient protein supply)
Peeling from sun exposure
White freckles
Halo nevi
Vitiligo
Post-inflammatory (atopic dermatitis, linear lichen sclerosis)
Post-infections (chickenpox, impetigo, insect bites, mycosis fungoides, pityriasis versicolor, tuberculoid leprosy)
Post-traumatic scars

**Table 2:** (a) and (b). Congenital diffuse cutaneous hypopigmentation disorders with neurological involvement.

Diffuse (a)	Localized (b)
Chediak–Higashi syndrome (CHS)	Waardenburg syndrome (WS)
Griscelli syndrome (GS)	Incontinentia pigmenti (IP)
Elejalde syndrome (ES)	Hypomelanosis of Ito (HI)
Cross syndrome (CS)	Tuberous sclerosis complex (TSC)
Tietz Albinism-Deafness Syndrome (TADS)	
Prader–Willi syndrome (PWS)	
Angelman syndrome (AS)	
Inborn errors of metabolism (phenylketonuria, histidinemia, homocystinuria, Menkes syndrome)	

## Data Availability

Data sharing is not applicable to this article because no new data was generated or analyzed in this study.

## Supplementary Materials

The following supporting information can be downloaded at: https://www.mdpi.com/article/10.3390/children12121621/s1, File S1: PRISMA 2020 Checklist.

## References

[1] Pinto F.J., Bolognia J.L. (1991). Disorders of hypopigmentation in children. Pediatr. Clin. N. Am..

[2] Cichorek M., Wachulska M., Stasiewicz A., Tymińska A. (2013). Skin melanocytes: Biology and development. Postepy Dermatol. Alergol..

[3] Yamaguchi Y., Hearing V.J. (2014). Melanocytes and their diseases. Cold Spring Harb. Perspect. Med..

[4] van Geel N., Speeckaert M., Chevolet I., De Schepper S., Lapeere H., Boone B., Speeckaert R. (2013). Hypomelanoses in children. J. Cutan. Aesthet. Surg..

[5] Tey H.L. (2010). A practical classification of childhood hypopigmentation disorders. Acta Derm. Venereol..

[6] Dessinioti C., Stratigos A.J., Rigopoulos D., Katsambas A.D. (2009). A review of genetic disorders of hypopigmentation: Lessons learned from the biology of melanocytes. Exp. Dermatol..

[7] Pavone P., Pappalardo X.G., Greco F., Parano C., Falsaperla R., Polizzi A., Ruggieri M. (2025). Congenital Localized Hypopigmentation Disorders as a Clue of Severe Neurologic Involvement: An Update Review. J. Child Neurol..

[8] Page M.J., McKenzie J.E., Bossuyt P.M., Boutron I., Hoffmann T.C., Mulrow C.D., Shamseer L., Tetzlaff J.M., Akl E.A., Brennan S.E. (2021). The PRISMA 2020 statement: An updated guideline for reporting systematic reviews. BMJ.

[9] Nagle D.L., Karim M.A., Woolf E.A., Holmgren L., Bork P., Misumi D.J., McGrail S.H., Dussault B.J., Perou C.M., Boissy R.E. (1996). Identification and mutation analysis of the complete gene for Chediak-Higashi syndrome. Nat. Genet..

[10] Ajitkumar A., Yarrarapu S.N.S., Ramphul K. (2025). Chediak-Higashi Syndrome. StatPearls.

[11] Al-Tamemi S., Al-Zadjali S., Al-Ghafri F., Dennison D. (2014). Chediak-Higashi syndrome: Novel mutation of the CHS1/LYST gene in 3 Omani patients. J. Pediatr. Hematol. Oncol..

[12] Pettit R.E., Berdal K.G. (1984). Chédiak-Higashi syndrome. Neurologic appearance. Arch. Neurol..

[13] Pastural E., Barrat F.J., Dufourcq-Lagelouse R., Certain S., Sanal O., Jabado N., Seger R., Griscelli C., Fischer A., Basile G.d.S. (1997). Griscelli disease maps to chromosome 15q21 and is associated with mutations in the myosin-Va gene. Nat. Genet..

[14] Ménasché G., Pastural E., Feldmann J., Certain S., Ersoy F., Dupuis S., Wulffraat N., Bianchi D., Fischer A., Le Deist F. (2000). Mutations in RAB27A cause Griscelli syndrome associated with haemophagocytic syndrome. Nat. Genet..

[15] Moradveisi B., Karimi A., Behzadi S., Zakaryaei F. (2021). Griscelli Syndrome in a seven years old girl. Clin. Case Rep..

[16] Haraldsson A., Weemaes C.M., Bakkeren J.A., Happle R. (1991). Griscelli disease with cerebral involvement. Eur. J. Pediatr..

[17] Elejalde B.R., Holguin J., Valencia A., Gilbert E.F., Molina J., Marin G., Arango L.A., Witkop C.J. (1979). Mutations affecting pigmentation in man: I. Neuroectodermal melanolysosomal disease. Am. J. Med. Genet..

[18] Duran-McKinster C., Rodriguez-Jurado R., Ridaura C., de la Luz Orozco-Covarrubias M., Tamayo L., Ruiz-Maldonando R. (1999). Elejalde syndrome—A melanolysosomal neurocutaneous syndrome: Clinical and morphological findings in 7 patients. Arch. Dermatol..

[19] Mohammadzadeh Shanehsaz S., Rezazadeh A., Dandashli A. (2015). Elejalde syndrome (ES). Dermatol. Online J..

[20] Afifi H.H., Zaki M.S., El-Kamah G.Y., El-Darouti M. (2007). Elejalde syndrome: Clinical and histopathological findings in an Egyptian male. Genet. Couns..

[21] Bahadoran P., Ortonne J.P., Ballotti R., de Saint-Basile G. (2003). Comment on Elejalde syndrome and relationship with Griscelli syndrome. Am. J. Med. Genet. Part A.

[22] Ivanovich J., Mallory S., Storer T., Ciske D., Hing A. (2001). 12-year-old male with Elejalde syndrome (neuroectodermal melanolysosomal disease). Am. J. Med. Genet..

[23] Cross H.E., McKusick V.A., Breen W. (1967). A new oculocerebral syndrome with hypopigmentation. J. Pediatr..

[24] Lerone M., Pessagno A., Taccone A., Poggi G., Romeo G., Silengo M.C. (1992). Oculocerebral syndrome with hypopigmentation (Cross syndrome): Report of a new case. Clin. Genet..

[25] Chabchoub E., Cogulu O., Durmaz B., Vermeesch J.R., Ozkinay F., Fryns J.P. (2011). Oculocerebral hypopigmentation syndrome maps to chromosome 3q27.1q29. Dermatology.

[26] Courtens W., Broeckx W., Ledoux M., Vamosa E. (1989). Oculocerebral hypopigmentation syndrome (Cross syndrome) in a Gipsy child. Acta Paediatr. Scand..

[27] Tezcan I., Demir E., Aşan E., Kale G., Müftüoğlu S.F., Kotiloğlu E. (1997). A new case of oculocerebral hypopigmentation syndrome (Cross syndrome) with additional findings. Clin. Genet..

[28] Tietz W. (1963). A syndrome of deaf-mutism associated with albinism showing dominant autosomal inheritance. Am. J. Hum. Genet..

[29] Izumi K., Kohta T., Kimura Y., Ishida S., Takahashi T., Ishiko A., Kosaki K. (2008). Tietz syndrome: Unique phenotype specific to mutations of MITF nuclear localization signal. Clin. Genet..

[30] Smith S.D., Kelley P.M., Kenyon J.B., Hoover D. (2000). Tietz syndrome (hypopigmentation/deafness) caused by mutation of MITF. J. Med. Genet..

[31] Ma V.K., Mao R., Toth J.N., Fulmer M.L., Egense A.S., Shankar S.P. (2023). Prader-Willi and Angelman Syndromes: Mechanisms and Management. Appl. Clin. Genet..

[32] Driscoll D.J., Miller J.L., Cassidy S.B., Adam M.P., Feldman J., Mirzaa G.M., Pagon R.A., Wallace S.E., Amemiya A. (1993). Prader-Willi Syndrome. GeneReviews^®^.

[33] Butler M.G., Miller J.L., Forster J.L. (2019). Prader-Willi Syndrome-Clinical Genetics, Diagnosis and Treatment Approaches: An Update. Curr. Pediatr. Rev..

[34] Takeshita E., Murakami N., Sakuta R., Nagai T. (2013). Evaluating the frequency and characteristics of seizures in 142 Japanese patients with Prader-Willi syndrome. Am. J. Med. Genet. A.

[35] Madaan M., Mendez M.D. (2025). Angelman Syndrome. StatPearls.

[36] Dagli A.I., Mathews J., Williams C.A., Adam M.P., Feldman J., Mirzaa G.M., Pagon R.A., Wallace S.E., Amemiya A. (1993). Angelman Syndrome. GeneReviews^®^.

[37] Zhou Q.J., Gong P., Jiao X.R., Yang Z.X. (2023). Clinical and molecular genetic analysis of Angelman syndrome with oculocutaneous albinism type 2: A case report and literature review. Beijing Da Xue Xue Bao Yi Xue Ban.

[38] Fridman C., Hosomi N., Varela M.C., Souza A.H., Fukai K., Koiffmann C.P. (2003). Angelman syndrome associated with oculocutaneous albinism due to an intragenic deletion of the P gene. Am. J. Med. Genet. A.

[39] Samanta D. (2022). Pharmacotherapeutic management of seizures in patients with Angleman Syndrome. Expert Opin. Pharmacother..

[40] Duis J., Nespeca M., Summers J., Bird L., Heus K.G.B., Valstar M.J., de Wit M.Y., Navis C., Hooven-Radstaake M.T., van Iperen-Kolk B.M. (2022). A multidisciplinary approach and consensus statement to establish standards of care for Angelman syndrome. Mol. Genet. Genomic. Med..

[41] Manssen L., Krey I., Gburek-Augustat J., von Hagen C., Lemke J.R., Merkenschlager A., Weigand H., Makowski C. (2025). Precision Medicine in Angelman Syndrome. Neuropediatrics.

[42] Stone W.L., Basit H., Los E. (2025). Phenylketonuria. StatPearls.

[43] Lam W.K., Cleary M.A., Wraith J.E., Walter J.H. (1996). Histidinaemia: A benign metabolic disorder. Arch. Dis. Child..

[44] Sacharow S.J., Picker J.D., Levy H.L., Adam M.P., Feldman J., Mirzaa G.M., Pagon R.A., Wallace S.E., Amemiya A. (1993). Homocystinuria Caused by Cystathionine Beta-Synthase Deficiency. GeneReviews^®^.

[45] Kaler S.G., DiStasio A.T., Adam M.P., Feldman J., Mirzaa G.M., Pagon R.A., Wallace S.E., Amemiya A. (1993). ATP7A-Related Copper Transport Disorders. GeneReviews^®^.

[46] Fujisawa C., Kodama H., Sato Y., Mimaki M., Yagi M., Awano H., Matsuo M., Shintaku H., Yoshida S., Takayanagi M. (2022). Early clinical signs and treatment of Menkes disease. Mol. Genet. Metab. Rep..

[47] De Feyter S., Beyens A., Callewaert B. (2023). ATP7A-related copper transport disorders: A systematic review and definition of the clinical subtypes. J. Inherit. Metab. Dis..

[48] Vairo F.P.E., Chwal B.C., Perini S., Ferreira M.A.P., de Freitas Lopes A.C., Saute J.A.M. (2019). A systematic review and evidence-based guideline for diagnosis and treatment of Menkes disease. Mol. Genet. Metab..

[49] Winters R., Masood S. (2025). Waardenburg Syndrome. StatPearls.

[50] Gowda V.K., Srinivas S., Srinivasan V.M. (2020). Waardenburg Syndrome Type I. Indian J. Pediatr..

[51] Chandra Mohan S.L.N. (2018). Case of Waardenburg Shah syndrome in a family with review of literature. J. Otol..

[52] Nusrat M., Tariq M.A., Aslam S., Zil-E-Ali A., Shahid M., Mahmood S. (2018). A Case of Waardenburg-Shah Syndrome Type 4 Presenting with Bilateral Homochromatic Blue Irises from Pakistan. Cureus.

[53] Khan T.A., Safdar C.A., Zameer S., Khushdil A. (2020). Waardenburg-Shah syndrome (WS type IV): A rare case from Pakistan. Perioper. Med..

[54] Scheuerle A.E., Ursini M.V., Adam M.P., Feldman J., Mirzaa G.M., Pagon R.A., Wallace S.E., Amemiya A. (1993). Incontinentia Pigmenti. GeneReviews^®^.

[55] Yadlapati S., Tripathy K. (2025). Incontinentia Pigmenti (Bloch-Sulzberger Syndrome). StatPearls.

[56] Meuwissen M.E.C., Mancini G.M.S. (2012). Neurological findings in incontinentia pigmenti; a review. Eur. J. Med. Genet..

[57] Ruggieri M., Polizzi A., Marceca G.P., Catanzaro S., Praticò A.D., Di Rocco C. (2020). Introduction to phacomatoses (neurocutaneous disorders) in childhood. Childs Nerv. Syst..

[58] Chamli A., Litaiem N. (2025). Hypomelanosis of Ito. StatPearls.

[59] Pavone P., Praticò A.D., Ruggieri M., Falsaperla R. (2015). Hypomelanosis of Ito: A round on the frequency and type of epileptic complications. Neurol. Sci..

[60] Ollech A., Hilewitz D., Tzadok M., Lahav E., Debby A., Eliyahu A., Barzilai A., Greenberger S. (2023). White epidermal nevus as an early sign of tuberous sclerosis complex-A case series. Pediatr. Dermatol..

[61] Randle S.C. (2017). Tuberous Sclerosis Complex: A Review. Pediatr. Ann..

[62] Hasbani D.M., Crino P.B. (2018). Tuberous sclerosis complex. Handb. Clin. Neurol..

[63] Curatolo P., Specchio N., Aronica E. (2022). Advances in the genetics and neuropathology of tuberous sclerosis complex: Edging closer to targeted therapy. Lancet Neurol..

[64] Northrup H., Aronow M.E., Bebin E.M., Bissler J., Darling T.N., de Vries P.J., Frost M.D., Fuchs Z., Gosnell E.S., Gupta N. (2021). Updated International Tuberous Sclerosis Complex Diagnostic Criteria and Surveillance and Management Recommendations. Pediatr. Neurol..

[65] Nair N., Chakraborty R., Mahajan Z., Sharma A., Sethi S.K., Raina R. (2020). Renal Manifestations of Tuberous Sclerosis Complex. J. Kidney Cancer VHL.

[66] Frudit P., Vitturi B.K., Navarro F.C., Rondelli I., Pozzan G. (2019). Multiple cardiac rhabdomyomas in tuberous sclerosis complex: Case report and review of the literature. Autops. Case Rep..

[67] Ruggieri M., Carbonara C., Magro G., Migone N., Grasso S., Tinè A., Pavone L., Gomez M.R. (1997). Tuberous sclerosis complex: Neonatal deaths in three of four children of consanguineous, non-expressing parents. J. Med. Genet..

[68] Gupta N., Henske E.P. (2018). Pulmonary manifestations in tuberous sclerosis complex. Am. J. Med. Genet. C Semin. Med. Genet..

[69] Hodgson N., Kinori M., Goldbaum M.H., Robbins S.L. (2017). Ophthalmic manifestations of tuberous sclerosis: A review. Clin. Exp. Ophthalmol..

[70] Specchio N., Nabbout R., Aronica E., Auvin S., Benvenuto A., de Palma L., Feucht M., Jansen F., Kotulska K., Sarnat H. (2023). Updated clinical recommendations for the management of tuberous sclerosis complex associated epilepsy. Eur. J. Paediatr. Neurol..

[71] Słowińska M., Kotulska K., Szymańska S., Roberds S.L., Fladrowski C., Jóźwiak S. (2021). Approach to Preventive Epilepsy Treatment in Tuberous Sclerosis Complex and Current Clinical Practice in 23 Countries. Pediatr. Neurol..

[72] Curatolo P., Nabbout R., Lagae L., Aronica E., Ferreira J.C., Feucht M., Hertzberg C., Jansen A.C., Jansen F., Kotulska K. (2018). Management of epilepsy associated with tuberous sclerosis complex: Updated clinical recommendations. Eur. J. Paediatr. Neurol..

[73] Jozwiak S., Curatolo P. (2021). Editorial: Tuberous Sclerosis Complex-Diagnosis and Management. Front. Neurol..

[74] Praticò A.D., Falsaperla R., Comella M., Belfiore G., Polizzi A., Ruggieri M. (2023). Case report: A gain-of-function of hamartin may lead to a distinct “inverse TSC1-hamartin” phenotype characterized by reduced cell growth. Front. Pediatr..

[75] Previtali R., Prontera G., Alfei E., Nespoli L., Masnada S., Veggiotti P., Mannarino S. (2023). Paradigm shift in the treatment of tuberous sclerosis: Effectiveness of everolimus. Pharmacol. Res..

[76] Nabbout R., Kuchenbuch M., Chiron C., Curatolo P. (2021). Pharmacotherapy for Seizures in Tuberous Sclerosis Complex. CNS Drugs..

[77] Śmiałek D., Kotulska K., Duda A., Jóźwiak S. (2023). Effect of mTOR Inhibitors in Epilepsy Treatment in Children with Tuberous Sclerosis Complex Under 2 Years of Age. Neurol. Ther..

[78] Curatolo P., Franz D.N., Lawson J.A., Yapici Z., Ikeda H., Polster T., Nabbout R., de Vries P.J., Dlugos D.J., Fan J. (2018). Adjunctive everolimus for children and adolescents with treatment-refractory seizures associated with tuberous sclerosis complex: Post-hoc analysis of the phase 3 EXIST-3 trial. Lancet Child Adolesc. Health.

[79] Auvin S., Baulac S. (2023). mTOR-therapy and targeted treatment opportunities in mTOR-related epilepsies associated with cortical malformations. Rev. Neurol..

[80] Thiele E.A., Bebin E.M., Filloux F., Kwan P., Loftus R., Sahebkar F., Sparagana S., Wheless J. (2022). Long-term cannabidiol treatment for seizures in patients with tuberous sclerosis complex: An open-label extension trial. Epilepsia.

[81] Sen A., Verner R., Valeriano J.P., Lee R., Zafar M., Thomas R., Kotulska K., Jespers E., Dibué M., Kwan P. (2021). Vagus nerve stimulation therapy in people with drug-resistant epilepsy (CORE-VNS): Rationale and design of a real-world post-market comprehensive outcomes registry. BMJ Neurol. Open.

[82] Specchio N., Pepi C., de Palma L., Moavero R., De Benedictis A., Marras C.E., Vigevano F., Curatolo P. (2021). Surgery for drug-resistant tuberous sclerosis complex-associated epilepsy: Who, when, and what. Epileptic. Disord..

[83] Ruggieri M. (2023). Neurologia Pediatrica-Dalle Cause Biologiche Alla Pratica Clinica.

[84] Zhou J., He X., Sun L., Xu J., Chen X., Chu Y., Zhou L., Liao X., Zhang B., Afvari S. (2024). Pre-trained multimodal large language model enhances dermatological diagnosis using SkinGPT-4. Nat. Commun..

[85] Boostani M., Bánvölgyi A., Zouboulis C.C., Goldfarb N., Suppa M., Goldust M., Lőrincz K., Kiss T., Nádudvari N., Holló P. (2025). Large language models in evaluating hidradenitis suppurativa from clinical images. J. Eur. Acad. Dermatol. Venereol. JEADV.

[86] Boostani M., Bánvölgyi A., Goldust M., Cantisani C., Pietkiewicz P., Lőrincz K., Holló P., Wikonkál N.M., Paragh G., Kiss N. (2025). Diagnostic Performance of GPT-4o and Gemini Flash 2.0 in Acne and Rosacea. Int. J. Dermatol..

